# Water Environment Assessment as an Ecological Red Line Management Tool for Marine Wetland Protection

**DOI:** 10.3390/ijerph14080870

**Published:** 2017-08-02

**Authors:** Yinan Zhang, Chunli Chu, Lei Liu, Shengguo Xu, Xiaoxue Ruan, Meiting Ju

**Affiliations:** College of Environmental Science and Engineering, Nankai University, Tianjin 300350, China; zhangyn2011@whu.edu.cn (Y.Z.); helenbooster@163.com (L.L.); xushengguo@mail.nankai.edu.cn (S.X.); ruan_nku@163.com (X.R.); jumeit@nankai.edu.cn (M.J.)

**Keywords:** water quality assessment, Qilihai wetland reserve, single factor evaluation, weighted average, water pollution, problems and suggestions

## Abstract

A ‘red line’ was established, identifying an area requiring for ecological protection in Tianjin, China. Within the protected area of the red line area, the Qilihai wetland is an important ecotope with complex ecological functions, although the ecosystem is seriously disturbed due to anthropogenic activities in the surrounding areas. This study assesses the water quality status of the Qilihai wetlands to identify the pollution sources and potential improvements based on the ecological red line policy, to improve and protect the waters of the Qilihai wetlands. An indicator system was established to assess water quality status using single factor evaluation and a comprehensive evaluation method, supported by data from 2010 to 2013. Assessment results show that not all indicators met the requirement of the *Environmental Quality Standards for Surface Water (GB3838-2002)* and that overall, waters in the Qilihai wetland were seriously polluted. Based on these findings we propose restrictions on all polluting anthropogenic activities in the red line area and implementation of restoration projects to improve water quality.

## 1. Introduction

Wetlands, which are natural ecosystems formed by interaction of land and water systems, with multiple ecological functions, are referred to as the “kidney of the Earth” [1]. Wetlands are an ideal habitat for many plants and animals, such as waterfowl, effectively providing a water source, controlling flooding and preventing soil desertification, as well as acting as a carbon sink, storing carbon and therefore reducing the greenhouse effect [2]. However, due to the fast pace of urbanization and industrialization in China, some critical ecological and environmental problems have emerged in regions such as the Qilihai wetlands in Tianjin, China [3]. The Qilihai wetlands, a core area of the Tianjin Ancient Coast and Wetlands National Nature Reserve (referred to as the Tianjin National Natural Reserve), is an area including the core area, buffer area and the experimental area, which is a marine nature reserve and home to various species such as East Asian-Australian migratory shorebirds [4]. Due to the overdevelopment of wetlands due to human expansion and anthropogenic activities, biological diversity has been reduced and the ecosystem services and other functions have degenerated [5], the aquatic environment has been polluted by intensive eutrophication, and the quality of water resources has decreased [6]. Due to the identification of serious levels of aquatic pollution, it is of critical importance to improve the water quality of the degraded Qilihai wetlands using regulatory controls and restoration projects [7] and prior to the implementation of these measures, it is necessary to analyze the Qilihai wetland water quality, to provide effective scientific strategies to protect wetland areas.

With continually increasing pollution of natural environments, the “ecological red line” strategy was promoted by the government of China in 2013 [8] in order to improve the quality of ecological environments and as an important tool for ensuring development of environmentally sustainable communities [9]. The “ecological red line” is an aggregate minimum area where development is strictly controlled, ensuring the sustainable provision of ecosystem functions, environmental quality and resource usage [10]. The application of the ecological red line management system to marine reserve management, has been a key step in the sustainable development of marine reserves in long term projects [11]. The ecological red line management approach can enhance regulatory systems for marine reserves, due to the inadequacies of current protection laws and regulations [12], protecting marine resources and ecological environments through compulsory policies. The “red line” system was explicitly established to protect important eco-function areas, eco-logically fragile areas and habitats for important species [13], based on a paid use system, where any company or individual who uses resources or affects the ecology, pays to compensate the damage [14]. A “red line” region was established in Tianjin, allowing the Tianjin National Natural Reserve to protect and improve its ecological function and water quality, as no development activities are allowed, as well as the policy stipulating that the water quality of the nature reserve should be managed in accordance with the highest standards of the *Environmental Quality Standards for Surface Water (GB3838-2002)*. As a protected natural reserve, the Qilihai wetlands are critical to the protection of regional natural resources, biological diversity and the integrity of the surrounding landscape [15,16]. The explicit regulations on water quality provided by red line policies, are a highly effective method to improve water quality with specific management systems and they should be enforced by local governments. Furthermore, it is essential to better understand wetland ecosystems, in particular their natural and environmental characteristics and diversity, prior to the implementation of corrective measures [17].

The Qilihai Wetland Reserve is rich in avian diversity, which is one of the most important components of the region’s biodiversity [18]. According to the Scientific Investigation Report on the Tianjin Ancient Coast and Wetlands National Nature Reserve Qilihai Wetland Core Area, 160 species of bird have been recorded, including three species belonging to those requiring first-class protection of Aves and 25 species requiring second-class protection of Aves. The flora types present in the Qilihai wetlands are also abundant, with 290 plant species present, with a rich compositional diversity of both wild and cultivated plants, such as crops, arbors and herbaceous communities, directly increasing the degree of fragmentation [19]. Additionally, the Qilihai wetlands are crucial for elements of urban construction, fishing, agriculture and other human activities such as recreation and tourism development [20].

Due to the increasing global population and rapid economic development, the demand for wetland resources is growing, along with continually increasing levels of deterioration and damage to surrounding ecosystems, creating an urgent environmental problem [21,22]. Much research has been undertaken, assessing the Qilihai wetlands ecosystems, with studies of the value of direct and indirect use of the Qilihai wetlands ecosystem [23,24], establishing that the ecological benefit of the Qilihai wetlands at the time of study, accounted for 72% of the total potential benefit (279.6 million US dollars), with consideration of the functional value in hydrology, water quality, erosion control, CO_2_ fixation and habitats [23]. Another study established an index system for the Qilihai wetlands to evaluate the status of ecosystems [25], while Xueping and Chen [26] used a 3D numerical model to study the water renewal capacity of the Qilihai Lagoon. Chen et al. [27] applied the same model to simulate the water environment and found problems due to water volume and pollution levels in Qilihai Lagoon [27]. The outcome of these research studies indicated the Qilihai wetlands ecosystem were under serious threat of biological diversity reduction and severe damage to oyster reefs and shell banks [28]. In addition, agricultural and industrial wastewater contains high concentrations of organic pollutants, heavy metals, petroleum, nitrogen and phosphorus, which accumulate in water and soil [29]. A further element to consider is that the increase in human land use and impact due to tourism, adds further pollutants into systems, which are transferred with the surface water and further drive regional pollution [30].

Based on the findings of these studies and current literature, there is much need to better assess the water quality of the Qilihai wetlands and find the pollution sources, before measures can be taken to protect the valuable wetlands due to the vast impact of anthropogenic activities on the Qilihai wetlands water quality. Additionally, the outcome of water quality assessment and protection proposals can assist decision making by managers and stakeholders, when planning systems to restore and improve the Qilihai wetlands ecosystem more efficiently. Moreover, this study tries to combine the ecological red line policy with water quality management, forming a plan to gradually prohibit all development activities in the Qilihai wetlands area, whilst improving and repairing water quality in accordance with the red line policy requirements. Based on previous research and monitoring data of water quality in the Qilihai Wetland Reserve, we establish an indicator system to analyze water pollution levels. We used the single factor evaluation method to analyze the compliance of each indicator and the Analytic Hierarchy Process (AHP) comprehensive evaluation method to evaluate the overall water quality in the Qilihai wetlands, over continuous monitoring years. Therefore, the aims of this study were as follows:

(a) To select exclusive indicators and construct an assessment system to assess water quality in the Qilihai Wetland Reserve;
(b) To perform out time series analysis from 2010 to 2013, to reflect the change in water quality in the Qilihai wetlands.
(c) To propose effective water quality management methods, according to the assessment results and the ecological red line regulations.

## 2. Materials and Methods

### 2.1. Site Description

**Figure 1:**
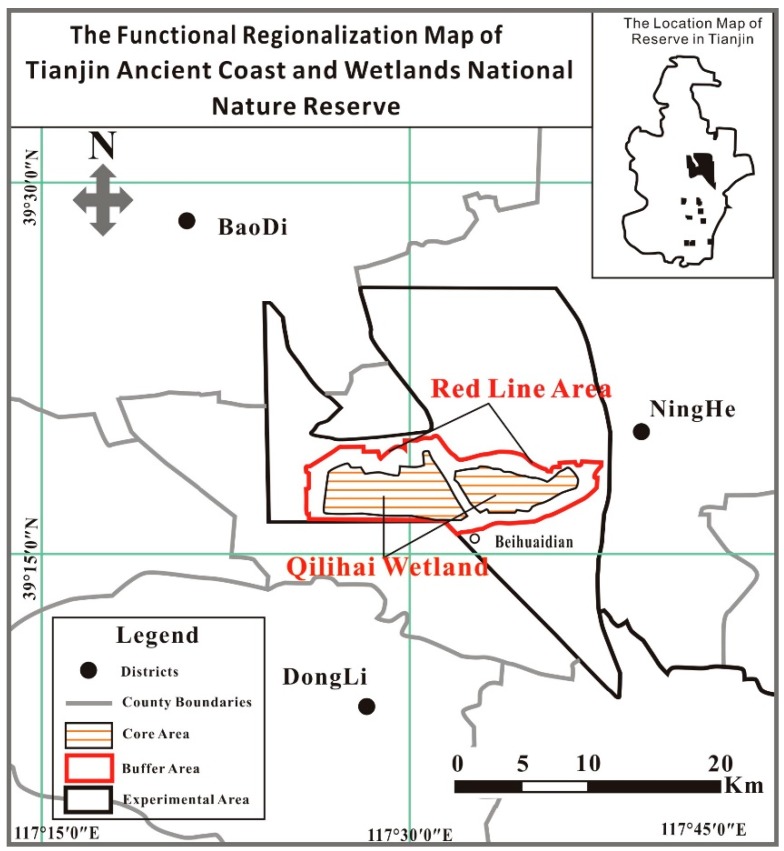
Map of the functional regionalization of the Tianjin Ancient Coast and Wetlands National Nature Reserve.

The Tianjin Ancient Coast and Wetlands National Nature Reserve (referred to as the Tianjin National Natural Reserve) is in the west bank of the Bohai Bay, in eastern Tianjin, China, which includes the districts of Ninghe and Jinnan, and part of the Binhai New Area. In 1992, the Tianjin Reserve was approved as a national nature reserve, representing the only national marine nature reserve with the mission of protecting and managing the natural environment and ecosystems, particularly the shell embankment, oyster reef of rare ancient coastal relics and wetlands areas. Tianjin National Natural Reserve belongs to a discontinuous, open protected reserve with an area of 359.13 km^2^, including the core area of 45.15 km^2^, a buffer area of 43.34 km^2^ and an experimental area of 270.64 km^2^. The Tianjin National Natural Reserve contains abundant natural resources, especially in the core area, with the functional regionalization of the Tianjin National Natural Reserve shown in Figure 1.

The Qilihai Wetland Reserve is situated in the core area of the Tianjin National Nature Reserve, and located in the southwest of Tianjin in the Ninghe District. The wetlands area is based around a 7000-year old ancient bay, which gradually evolved into an ancient-lagoon-type wetland. The Qilihai wetlands belong to Chaobai New River Basin with an actual storage capacity of 300 million m^3^, which attracted tourist destination and support important agricultural and fishing economies with 53 km^2^ of arable land and 16 km^2^ of aquaculture water surface area [31]. It is divided into two parts by the Chaobai New River, also adjacents to Zengkou River in the north, Jintang river in the south, connects Jiyun river in the east [32]. Qilihai wetland is a natural reservoir which provides water for surrounding rivers [33]. The underground aquifer is between 50 and 70 m, and the chemical type of groundwater is Na-HCO_3_ [34]. The Qilihai wetlands are surrounded by five townships, 25 administrative villages, and have a population of some 87,000, with most of the population relying heavily on wetland resources for various elements of survival, such as food, water and to make a living. Unfortunately, industry, agricultural production and urban construction in the region have significantly adversely affected marine ecosystems in the wetlands, as well as depleting many essential resources.

**Figure 2:**
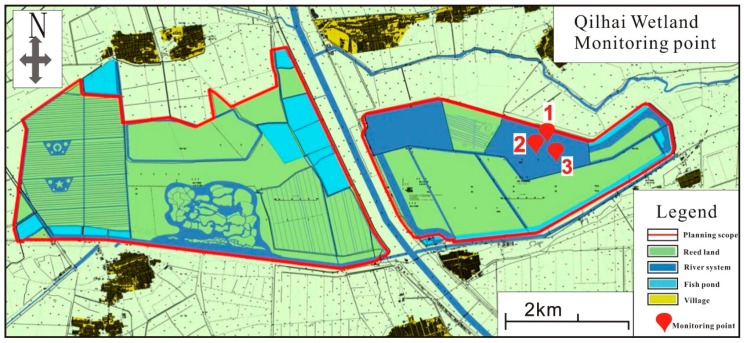
The map of monitoring sites in the Qilihai Wetland. Numbers 1, 2 and 3 refer to the monitoring points.

Three monitoring sites were established around Bird Island to investigate water quality, an important island for large bird species located in the core area of the Qilihai wetlands. Bird Island is surrounded by large body of water (Figure 2) and is a popular tourism site due to the native birds, resulting in pollution in the surrounding water due to large levels of rubbish dropped by tourists and discharged wastewater, more detail information can be seen in Table 1. In addition, a large quantity of farms are located on the north of Bird Island, with eutrophication being driven by the continually increasing levels of a large number of fertilizers, nitrogen and phosphorus in the water [35]. Therefore, the data collected at monitoring points will reflect the water quality of the Qilihai wetlands, allowing assessment of the influenced of anthropogenic activities. Data was provided by the Tianjin Ancient Coast and Wetland National Nature Reserve Management Office, an administrative organization responsible for continual monitoring of ecological environments and are also responsible for the daily management of the Tianjin National Natural Reserve. The specific location of monitoring sites is shown in Figure 2.

### 2.2. Analytical Method

#### 2.2.1. The Indicator System

Most studies use physical, chemical and biological characteristics to evaluate water quality and pollution status [36], such as dissolved oxygen (DO), pH, suspended substances (SS), transparency and temperature, among others, as physical characteristics [35,37]; total phosphorus (TP), total nitrogen (TN), ammoniacal nitrogen (NH_3_-N), chemical oxygen demand (COD) [38], biochemical oxygen demand (BOD), petroleum, and heavy metal, among others, as chemical characteristics [39,40]; and chlorophyll a, biomass of benthos and diversity of rare species, among others, as biological characteristics [41,42]. These indicators are commonly selected in studies utilizing the evaluation index system for water quality assessment; therefore, in order to comprehensively assess the water quality of Qilihai Wetland Reserve, all eight pollution indicators were selected for time series analysis in samples from the Qilihai wetland core area, between 2010 and 2013. Water quality indicators are divided into three categories, which include general indicators, pollution indicators and biological indicators. General indicators are the pH, dissolved oxygen and transparency, which represent the general physical and chemical properties of the Qilihai wetlands aquatic environment. COD [38], TN, TP and petroleum (Pet) were selected as pollution indicators to establish the extent and degree of water pollution. TN and TP represent the degree of eutrophication in the water body, which plays an important role in water quality in the Qilihai wetlands [34], while petroleum is a common pollutant which has been found to affect the growth of various species at different levels of the ecosystem, such as reeds and fish and is sourced to industrial activity and fishing vessel leakage in the region [43]. The chlorophyll a (Chl-a) reflects the growth of phytoplankton [44], so is therefore used as a biological indicator to examine the effect of environmental factors on the primary productivity in this aquatic system. The samples were collected and analyzed of the monitoring points by professionals from the Tianjin Ancient Coast and Wetland National Nature Reserve Management Office, and the analytical method is shown in Table 2.

#### 2.2.2. Assessment Method

Effective water quality management requires a thorough understanding of pollutant sources, transport and fate, but complex monitoring data rarely provides a simple outcome [45], so mathematical methods are applied to interpret water quality data effectively [46]. This study evaluates the water quality in the Qilihai Wetland Reserve environment using single factor and comprehensive assessment methods. Single factor assessment is a simple method which compares the measured value of the index, with the water environment quality standards, to interpret whether the monitoring factor meets standard requirements. Xianbin Liu et al. [47] used the single factor method to analyze the water quality of the Haihe River basin, with results showing that the main pollutants were total nitrogen, ammoniacal nitrogen [48,49], COD and BOD [47]. *The Environmental Quality Standards for Surface Water (GB3838-2002)* are the national regulatory environmental quality standards, with established limit values for water quality environmental indicators in the National Nature Reserve. Therefore, this study applies *GB3838-2002* as the evaluation criteria.

Single factor evaluation is a relatively simple and established method in water quality assessment [50], although it commonly overestimates or underestimates the degree of water pollution since the class of water quality is determined only on the basis of the worst indicator [51]. Therefore, the need for a comprehensive evaluation of overall water quality is necessary, integrating complex data and generating a score for the purpose of efficient continual assessment and interpretation of the water quality status [52]. Additionally, the weighted sum method is an established evaluation method which obtains an overall score based on the individual indices [53]. Almost all of the water quality indicators included in the index should be normalized according to the expected concentration, to allow scaling and interpretation of the good or bad water quality [54]. The parameter values are weighted according to their importance to the overall water quality [55] and the index is calculated by the weighted average method [56,57,58]. Weights were assigned to all indicators and the water quality pollution index (PI) was calculated by the weighted average method (as expressed by formula (1)). The distribution of weights to the multi-criteria is the key step in evaluation, while according to [59], the methods for determining weights can be divided into two categories: the subjective method, which includes applications such as the Fuzzy Comprehensive Evaluation Method [60] and Analytic Hierarchy Process (AHP) [61], which involve calculating weights by comprehensive consultation; and the objective method, which includes Factor Analysis [62], TOPSIS [63] and Principal Component Analysis [59], among others, determine weights according to the interrelationship between indicators or the indicators variation level. Although objective methods are convenient to use and consider more factors, the results cannot indicate the relative importance of different factors [64]. Therefore, the AHP method was selected to calculate the weight of each indicator. AHP, which combines the qualitative and quantitative analysis in order to provide better analysis to decision-makers [65] was put forward by the American operational research expert Saaty in the 1970s [66]. Through the calculation of the experts in the field of the environment, the weight is determined, and can reflect the degree of importance of each indicator [67]. AHP compares each of the two indicators, and the scores are assigned according to their degree of importance. The score 5 to 1 means absolute important, very important, relative important, moderate important and general important, and weight is calculated through the matrix model respectively.

The water quality PI represents the water quality in the Qilihai wetlands and is defined by Equation (1):

(1) $$PI=\sum_{i=1}^{n}W_{i}\frac{C_{i}}{C_{oi}}$$

where *Wi* refers to weight of each index; *C_oi_* is the standard value of every indicator as advised by the *Environmental Quality Standards for Surface Water (GB3838-2002);* and the ratio of *C_i_* and *C_oi_* is the process of standardization. The weight of each index was calculated by the AHP method with the professional opinions considered, including input from the Tianjin National Natural Reserve, academic institutions and communities working or living around the Qilihai wetlands. To establish the most important effect of environmental aquatic pollution, based on its impact on the Qilihai wetlands, we set the weight of indicators COD, TN, TP, and chlorophyll a, to be higher than other indicators, with all weights listed in Table 4, where C_i_ represents the monitoring value of every indicator, as provided by the Wetland National Nature Reserve Management Office, and we also listen to their suggestions when we determined the degree of importance of each indicator, as well as calculating the weights. It is of note, that the reference does not contain the standard value for chlorophyll a or transparency, the final confirmation of which depended on a wide number of academic studies and international standards. Annual variation is observed in water quality, so results were calculated to three levels using the standard deviation classification method (Table 3), which utilizes the smallest PI value as the graded endpoint ($PI_{min}$) and the range of pollution is from $PI_{min}$ to the sum of $PI_{min}$, while the standard deviation (SD) refers to the variation in PI measurements during 2010–2013.) ($PI_{min}$ ~ $PI_{min}+SD$), with a smaller acceptable range during moderate pollution events ($PI_{min}+SD$ ~ $PI_{min}$ + 2 × *SD*), as compared to severe pollution events $(PI_{min}+2\times SD$ ~ $PI_{min}$ + 3 × *SD*).

## 3. Results

### 3.1. Water Quality Based on Specific Indicators

**Figure 3:**
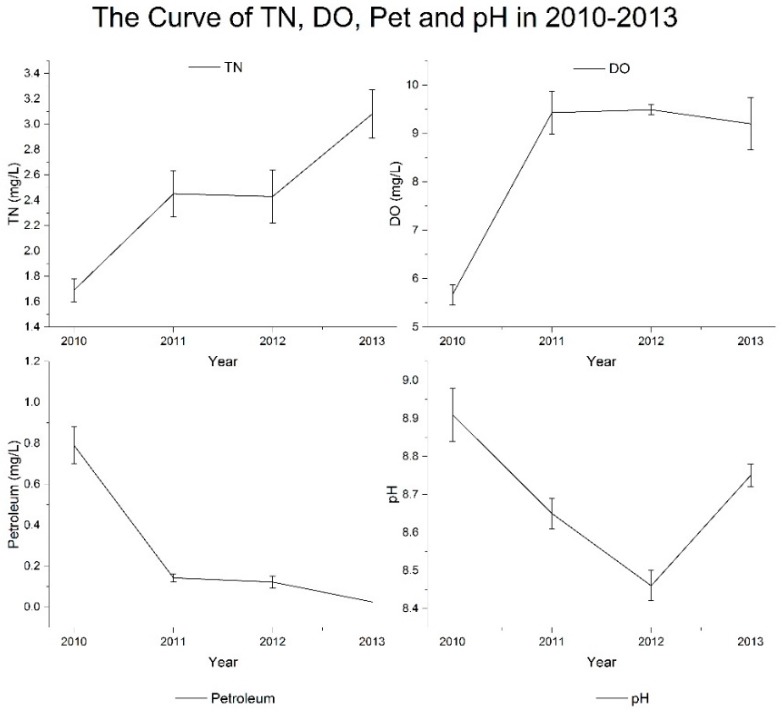
The average concentration curves of the indices total nitrogen (TN), dissolved oxygen (DO), petroleum (Pet) and pH between 2010 and 2013.

**Figure 4:**
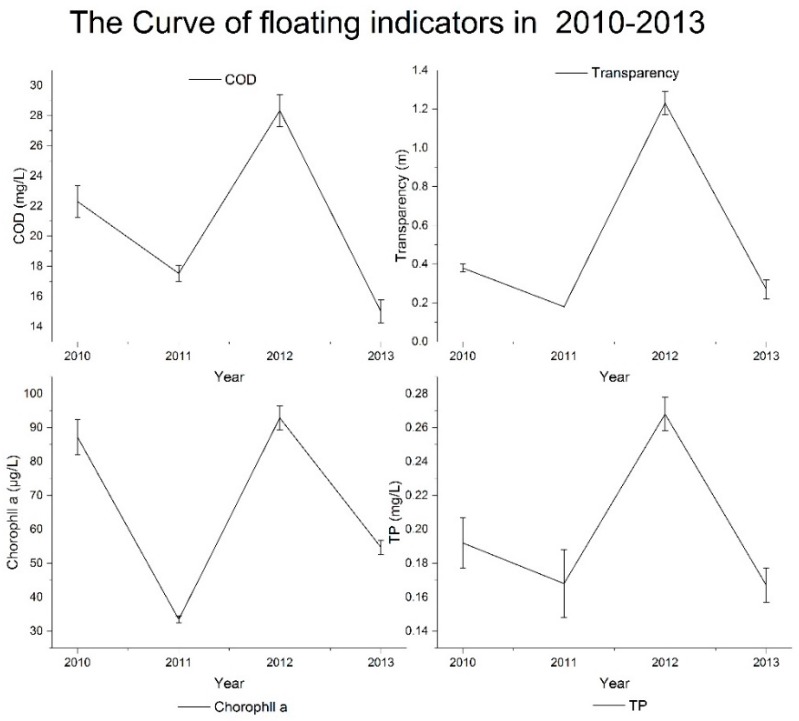
The average concentration curves of floating indicators from 2010 to 2013.

**Figure 5:**
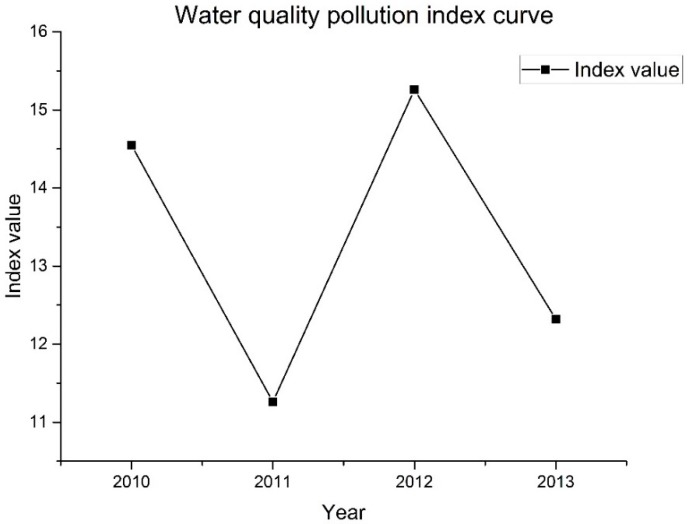
The water quality pollution index curve.

The motoring values, standard values and the weight of each indices, is presented in Table 4. As the *Environmental Quality Standards for Surface Water (GB3838-2002)* outline regulations on water quality, indicators at the national nature reserve should reach the fist-class standard values for surface water quality. The results show that some water quality indicators at the Qilihai Wetland Reserve do not meet the standard requirement, reflecting the low water quality overall in the wetland area. The variation curves of all indicators between 2010 and 2013 are shown in Figure 3, Figure 4 and Figure 5.

The dynamics of all factors observed in the monitoring years, can be divided into four types, including deteriorative, floating, stable or improving trends. Firstly, TN showed a deteriorative trend (Figure 3), where TN concentrations exhibited a significant upward trend from 2010 to 2013, with values increasing from 1.69 mg/L to 3.08 mg/L.

This in turn accelerates the degree of eutrophication in the aquatic environment, reducing water quality and resulting in a reduction in dissolved oxygen and inducing toxicity in aquatic organisms, such as free ammonia toxicity to fish species. It is of note, that TN did not meet the requirements set by the *Environmental Quality Standards for Surface Water (GB3838-2002)*, where the pollution level was established to be below class V, defined as a high level of TN pollution that affects water quality seriously. These findings show that the regulators of the Qilihai wetlands, should make every effort to strictly control the concentration of TN and take immediate measures to improve the water quality.

In the analysis, we identified significant variation in some indicators during the monitoring period, including the indicators TP, COD, Chlorophyll a and transparency (Figure 4). An unstable water quality environment can result in the outbreak of water pollution incidents, for example, the concentration of TP in 2012 was 0.268 mg/L, higher than the years 2010 and 2011 by 39.6% and 59.5%, respectively, with neither of these years meeting the water quality standards for TP concentrations.

TP pollution increased further in 2012, with the pollution level, then decreasing from class III to class IV, resulting in the concentration of TP in 2013 being reduced by 13.02% as compared to 2012, showing the TP control measures implemented in 2013 achieved a significant result. In 2011, the COD concentration was 17.53 mg/L, which decreased by 21.4% as compared to 2010; then suddenly rose again in 2012 by 61.6% as compared to 2011; and reduced again significantly by 47.1% in 2013, reaching the water quality standard class I. Overall, an increasing trend in COD was observed and although significant variation was observed annually, the water quality related to organic pollution in the Qilihai wetlands, is improving. The dynamics in chlorophyll a concentration, followed the same pattern as that of COD, where the concentration of chlorophyll a in 2010 was 0.087 mg/L, decreasing in 2011 by 61.6%, then increasing again in 2012 with concentrations of 0.093 mg/L, then finally decreasing in 2013 by 41.1%. Moderate chlorophyll a content in water means a good integrity and health status of phytoplankton communities. But the content of chlorophyll a in the Qilihai Wetland Reserve seriously exceeded the standard, indicating deterioration of the ecological flora environment, driving further eutrophication of the water body. The transparency indicator refers to the degree of clarity of the water, the lower value indicates more suspended and colloidal particles in the water. The highest transparency value observed during the monitoring period was 1.23 m in 2012, lower than the minimum transparency outlined in the regulatory standard, while transparency values of other years reached no more than 0.4 m. The transparency of water in the Qilhai wetland reserve reflects the poor physical properties of the aquatic environment.

Compared with other indices, pH was the most stable during the monitoring period showing no significant change (Figure 3), as well as successfully meeting water quality standards throughout. The water environment was weakly alkali, which has been found to be conducive to the growth of aquatic organisms. Among the many indicators investigated, only DO and the petroleum index showed an overall trend of improvement (Figure 3). When the concentration of DO, referring to dissolved molecular oxygen, reduces below 5 mg/L, harmful effects are induced on aquatic organisms, such as difficulty in breathing for some fish species. The monitored concentration of DO in 2013 was 9.2 mg/L, which was similar to the data from 2011 and 2012, although higher by 70% than 2010. The DO concentration reached the standard value in 2011 and maintained this level since, indicating that the amount of DO in the Qilihai wetlands is sufficient for survival. The petroleum pollution level was found to decline gradually from 2010 to 2013, with the pollutants concentration in 2013 meet the water quality standard, following reduction since 2010, 2011 and 2012, by 96.8%, 82.5% and 79.5%, respectively. The dynamics of both DO and petroleum pollution reflected that the water quality of the Qilihai wetland area core areas was gradually improving overall.

### 3.2. Overall Water Quality Assessment

The water quality PI was calculated to evaluate water quality at the Qilihai Wetland Reserve, established based on the weighted average of individual indicators. The PI curve from 2010 to 2013 showed the variations in pollution levels during the monitoring period (Figure 5), with the water pollution degrees classified into three levels and a standard deviation value of 1.62.

The range 11.2~12.8 represents areas where the Qilihai wetlands were slightly polluted; values of 12.8~14.5 represent areas where the Qilihai wetland was moderately polluted; and values of 14.5~16.2 indicate areas where the Qilihai wetland was severely polluted. Classification results showed that 2011 and 2013 showed only slightly polluted levels, while the years 2010 and 2012 showed severe pollution levels (Table 5), showing continual fluctuations between levels of moderate and severe pollution. The reduced water quality observed were mainly due to pollution caused by TP, TN and chlorophyll an indicators. Although TP, TN, transparency and chlorophyll a showed significant disparity as compared to the standard value, such as TP concentrations being 20-fold higher than the standard, causing the PI value to be higher than 1 (representing the level where all indicators reach the established standard values). As a result, the overall water quality situation of the Qilihai Wetland Reserve can be considered as serious. The water quality pollution index curve for the Qilihai Wetland Reserve fluctuates significantly, with recorded PI values in 2011 and 2013 of 11.26 and 12.32, respectively, which were notably lower than the values obtained for 2010 and 2012. The unstable status of the overall water quality of the Qilihai Wetland Reserve, resulted in the fluctuation of some pollution indicators and biological indicators, such as TP, COD and chlorophyll a. As both the pollution and biological indicators had a profounder influence on water pollution, the weights of these indicators were set higher than those for the general indicators. Therefore, due to the high degree of variation in data, the assessment results showed an unsatisfactory water quality in the Qilihai Wetland Reserve, supporting evidence that it is both necessary and urgent to progress plans for improvement of the aquatic environment in this region.

## 4. Discussion

The water quality for the Qilihai Wetland Reserve was assessed using various indicators, where the concentration of TN showed a significant upward trend, highlighting the need for strict TN control measures. Fluctuations in chlorophyll a transparency, COD and TP values during the monitoring period, suggest an unstable phytoplankton environment, which is another critical area requiring future improvement. The assessment of DO and petroleum pollution showed an improving trend, with organic pollution levels generally exhibiting a declining trend. The observed pH values were steady, meeting the water quality standard requirements. It is of note that some ecological restoration measures have improved some aspects of water quality, such as DO and the reduction of petroleum related and organic pollutants [68]. Despite this, the level of water pollution is still critical, especially with regards to TN and TP, meaning more strict treatment methods, management and control measures are needed to reduce the level of nitrogen and phosphorus supplied by agricultural fertilizer runoff. A new sustainable model for residential activity and tourism development is critical for the protection of aquatic environments and to improve the ecological environment of the Qilihai reserve [69]. Classification is helpful to reflect the different grade of water quality while evaluating the overall pollution situation and the outcomes of this classification model indicated that the entire aquatic environment was polluted to varying degrees during the monitoring period, while the results show an overall floating trend.

Results showed that the overall level of water pollution at the Qilhai Wetland Reserve was quite serious, with the key driving factors identified as:

1. *Emissions of large amounts of pollutants*: Most villagers in the vicinity of the wetlands area rely on aquaculture and fisheries for a living, therefore pesticides, fertilizers and farming cause significant levels of non-point source pollution [70]. These pollution sources and domestic sewage are all discharged into wetland waters, causing COD, TP, TN and other indicators to increase significantly, which also increases the degree of eutrophication [28]. Despite the economic interests in tourism development for the local residents, high levels of tourism and excessive visitors levels, results in a material and energy load beyond the capacity of the systems in the area, resulting in a transfer of metabolites generated by anthropogenic activities being continually applied to the Qilhai wetland system, therefore destroying the aquatic environment [33]. In addition, agriculture, fisheries, tourism and villages have greatly occupied the wetland area, especially the fish pond, obstructing circulation between water resources, and reducing the self-purification capacity of the wetlands ecosystem [71].
2. *Lack of comprehensive management tools*: Firstly, despite many laws and regulations related to nature reserves, such as the red line policy, relevant regulations are still lacking for the ancient coastal wetlands, ensuring implementation of these protection policies [72]. Secondly, the control and supervision of industrial, agricultural, fishery and tourism pollution sources are inadequate, leading to an excessive discharge of pollutants [73]. On the other hand, it is difficult to control pollution in the Qilihai Natural Reserve due to the decentralized protection areas and the lack of protection facilities and infrastructure [74]. Moreover, there is a lack of awareness among local residents regarding environmental wetland protection [75], as residents are not aware of the ecological value of the protected areas, nor do they know how to avoid damaging activities in the protection zone.

These results reveal an objective assessment of the characteristics of water quality in protected areas, which in this case calls for strengthening of water quality management systems in the Qilihai wetlands. Firstly, statutory regulations should prohibit any anthropogenic activities that are unrelated to protection within the red line area, which includes the core area (the Qilihai wetland) and the buffer area of the Tianjin National Natural Reserve. As there are still a large number of residents living within the buffer area, regulations should forbid all fishing, agriculture and tourism in the core area initially, then move the dispersed interior inhabitants and associated production activities within the buffer area out to the intensively constructed eco-town [76]. Secondly, the Tianjin Qilihai Wetland Park tourism area should be moved to the experimental area and developed as an ecological tourism area, with a beautiful natural environment that is ideal for ecological education with the purpose of environmental protection. Following the field survey, we found that the Tianjin Qilihai Wetland Park has prohibited visitors in the recent month prior to sampling, indicated that the Qilihai wetland environment needed recover and repair to a level serious enough to attract governmental attention. Moreover, there is a need to establish a continuous monitoring system for long-term dynamic monitoring and water environment assessment, allowing immediate water quality interpretation at all times [77].

In order to improve the water quality of the Qilihai wetlands, control measures must target the pollutant sources, following identification through source apportionment analysis [78]. This allows the development of remediation measures to improve water quality using technical means such as ecological restoration, dredging engineering and non-point source pollution control [79]. We propose that it is necessary to restore water quality using systems optimized according to the wetland type and water characteristics [80], resulting in more effective outcomes in symptomatic treatments. Subsequent protection projects should then be performed in order to meet the basic principles of the red line policy and to ensure the level of ecological function does not decrease [81]. In addition, statutory regulations should integrate local regulation with wetland ecology and sustainable management [82], to help promote and protect the wetland area more forcefully and effectively. Development of infrastructure and protection facilities are required, to protect the nature reserve [83] and to devote more efforts to education and public awareness, encouraging local communities to become active in environmental protection. When there is conflict between protection measure and residents’ interests, compensation should be provided to the residents or companies that are affected by environmental protection precedence. Moreover, to enhance water quality in the long term, the government and stakeholders should recover and protect environment quality using management tools, the findings of scientific research and technology projects.

It should be noted that this study has some limitations. Firstly, the number of indicators could be increased appropriately, such as the heavy metal index, so that the results can be analyzed comprehensively. Also, the proposals for improvement presented in this article are unilateral and further quantity analysis should be performed to find the mechanism(s) of pollution and establish detailed pollution control method proposals. Despite the preliminary nature of this study, it clearly indicates a flexible method to assess water quality in nature reserves, based on selection of the appropriate index system according to specific research purposes. In addition, we present proposals, based on the ecological red line policy, to protect the Qilihai wetlands more effectively. Overall these findings show that it is necessary to assess the Qilihai wetland eco-system, as the ecological environment is becoming increasingly vulnerable, resulting in a need for extended research about ecological evaluation indices, as a tool to measure ecological environment quality and the Qilhai wetland reserve is an ideal environment for this analysis.

## 5. Conclusions

In this study, an assessment system is established for the assessment of the water quality status, supported by monitoring data from between 2010 to 2013. The situation and time-series dynamics for all indicators were analyzed firstly by a single factor evaluation method; and the overall water quality of Qilihai Wetland Reserve and time-dependent transformation during the monitoring period was explored using a comprehensive evaluation method; finally, we summarized drivers of the low water quality and make some proposals for improvements. Result show that the overall water pollution was quite serious, mainly affected by the indicators of TN, TP, COD, chlorophyll-a. Therefore, pollution should be controlled from these indicators with more targeted and organized water quality management. Furthermore, there is a balance between sampling quantity and trend analysis. We rely on comprehensive analysis methods and simple data to reflect the water quality trend of the Qilihai wetlands, instead of a quite large number of sample data and statistical analysis. Therefore, we were more focused on the importance of data quality, which was based on data provided by the Tianjin Ancient Coast and Wetland National Nature Reserve Management Office that is scientifically analyzed.

The water pollution in Qilihai was found to be caused by the various pollutants which are emitted into the Qilihai wetlands, due to weaknesses in the statutory regulations and management systems in the region. Therefore, management and remediation processes are required to improve water quality in the Qilihai Wetland Reserve, based on its specific water characteristics. Regulatory bodies and governments should strictly comply with the requirements of the ecological red line, ensuring the water quality meets required standards. Moreover, the residents should cooperate with statutory schemes and projects to protect the Qilihai wetland environment, as wetland reserves are of great importance to the ecological red line policy to protect wetland ecosystems combined with local environment management. Prior to implementation of ecological protection policies, it is necessary to better understand the current situation of ecological environments such as presented in this paper, to avoid inconformity between policies and ecosystems and to improve management efficiency.

## Figures and Tables

**Table 1:** Sampling sites layout information of Qilihai Wetland Core Area.

Monitoring Points Number	Longitude	Latitude
1	117°36′00.0′′	39°18′06.20′′
2	117°36′0.12′′	39°17′57.66′′
3	117°36′2.51′′	39°17′47.96′′

Numbers marked around the monitoring points correspond to the monitoring points number in Table 1.

**Table 2:** The analytical method of each indicator.

Indicator	Analytical Method	Method Source
pH	Glass electrode method	GB 6920-86
DO	Iodometric method	GB7489-87
COD	Dichromate method	GB11914-89
TN	Alkaline persulfate digestion UV spectrophotometry	GB11894-89
TP	Ammonium molybdate spectrophotometric method	GB11893-89
Petroleum	Infrared spectrophotometry	GB/T16488-1996
Chlorophyll a	Spectrophotometry	SL88-2012
Transparency	Seine disc method	---

DO: dissolved oxygen; COD: chemical oxygen demand; TN: total nitrogen; TP: total phosphorus.

**Table 3:** The scale and error margins of various grades of the pollution index (PI).

Classification	Value Range
Slight pollution	$PI_{min}$ ~ $PI_{min}+SD$
Moderate pollution	$PI_{min}+SD$ ~ $PI_{min}$ + 2 × *SD*
Severe pollution	$PI_{min}+2\times SD$ ~ $PI_{min}$ + 3 × *SD*

SD: Standard deviation.

**Table 4:** Indicator data used to establish the water quality index.

Index Category	Index	Motoring Value				Standard Value	SD	Weight
		2010	2011	2012	2013			
**general indicator**	pH	8.91	8.65	8.46	8.75	6-9	0.16	0.036
	DO	5.67	9.43	9.49	9.20	7.5	1.61	0.107
	transparency	0.38	0.18	1.23	0.27	5	0.42	0.107
**pollution indicator**	COD	22.30	17.53	28.33	14.99	15	5.08	0.178
	TN	1.69	2.45	2.43	3.08	0.2	0.49	0.178
	TP	0.192	0.168	0.268	0.167	0.01	0.04	0.178
	petroleum	0.79	0.143	0.122	0.025	0.05	0.30	0.059
**biological indicator**	chlorophyll a	0.087	0.034	0.093	0.055	2 × 10^−3^	0.024	0.157

All index measured in mg/L. Transparency measured in meter.

**Table 5:** Overall water quality assessment outcomes.

Year	Value	Classification	Range
2010	14.55	Severe pollution	14.5–16.2
2011	11.26	Slight pollution	11.2–12.8
2012	15.26	Severe pollution	14.5–16.2
2013	12.32	Slight pollution	11.2–12.8
